# Adalimumab Therapy Improves Intestinal Dysbiosis in Crohn’s Disease

**DOI:** 10.3390/jcm8101646

**Published:** 2019-10-09

**Authors:** Davide Giuseppe Ribaldone, Gian Paolo Caviglia, Amina Abdulle, Rinaldo Pellicano, Maria Chiara Ditto, Mario Morino, Enrico Fusaro, Giorgio Maria Saracco, Elisabetta Bugianesi, Marco Astegiano

**Affiliations:** 1 Department of Surgical Sciences, University of Turin, 10124 Turin, Italy; mario.morino@unito.it; 2 Department of Medical Sciences, University of Turin, 10124 Turin, Italy; amina.abdulle@edu.unito.it (A.A.); giorgiomaria.saracco@unito.it (G.M.S.); ebugianesi@yahoo.it (E.B.); 3 Unit of Gastroenterology, Molinette Hospital, 10126 Turin, Italy; rinaldo_pellican@hotmail.com (R.P.); marcoastegiano58@gmail.com (M.A.); 4 S.C. Reumatologia, Città della Salute e della Scienza di Torino, 10126 Turin, Italy; mariachiaraditto@gmail.com (M.C.D.); fusaro.reumatorino@gmail.com (E.F.)

**Keywords:** *Bacteroides ovatus*, *Bifidobacterium adolescentis*, Dysbiosis, *Faecalibacterium prausnitzii*, *Ruminococcus gnavus*

## Abstract

The response to treatment with biologic drugs, in patients with Crohn’s disease, could be associated with changes in gut microbiota composition. The aim of our study was to analyse the modification of microbiota during adalimumab therapy in patients with Crohn’s disease. We performed a prospective study in patients with Crohn’s disease analysing gut microbiota before start of adalimumab therapy (T0) and after six months of therapy (T1). Among the 20 included patients, the phylum * Proteobacteria* fell from 15.7 ± 3.5% at T0 to 10.3 ± 3.4% at T1 (*p* = 0.038). Furthermore, the trend in relation to therapeutic success was analysed. Regarding bacterial phyla, *Proteobacteria* decreased in patients in whom therapeutic success was obtained, passing from a value of 15.8% (± 4.6%) to 6.8 ± 3.1% (*p* = 0.049), while in non-responder patients, percentages did not change (T0 = 15.6 ± 5.7%, T1 = 16.8 ± 7.6%, *p* = 0.890). Regarding the *Lachnospiraceae* family, in patients with normalization of C reactive protein six 6 months of adalimumab therapy, it increased from 16.6 ± 3.1% at T0 to 23.9 ± 2.6% at T1 (*p* = 0.049). In conclusion, in patients who respond to Adalimumab therapy by decreasing inflammation, there is a trend of intestinal eubiosis being restored.

## 1. Introduction

Inflammatory bowel diseases (IBD) are chronic diseases that share immune-mediated pathogenesis and relapsing course [1]. Crohn's disease (CD) and ulcerative colitis (UC) are the two main IBD types. The exact pathogenesis of IBD remains unknown. The most recent studies agree in identifying an individual genetic susceptibility strongly conditioned by environmental factors and by the interaction between intestinal microbiota and the body's immune response [2,3]. Changes in the epidemiology of IBD over time and in different geographical areas suggest that environmental factors play an important role in inducing or modifying the expression of the disease [4]. Considering that IBD emerged in Western countries around the middle of the 20th century and the increased incidence of IBD in developing countries over the last 25 years, this epidemiological evolution is supposed to be linked to both the Westernization of the lifestyle and industrialization. Urbanization is associated with dietary changes, antibiotic use, hygienic status, microbial exposure and pollution, all implicated as potential environmental risk factors for IBD [5]. A consequence of Westernization of the lifestyle seems to be dysbiosis, defined as a loss of diversity of composition of microbiome in an individual. Microbial diversity decreases in patients with CD compared to subjects without CD [6].

Biological drugs, first of all anti-tumor necrosis factor (TNF), are able to modify the natural history of numerous inflammatory diseases [7], in part by acting directly on inflammation and partly indirectly with mechanisms not yet fully understood. Few studies have analysed the effect of adalimumab therapy on specific bacteria of intestinal microbiota in adult IBD patients [8].

The aim of our study was to analyse microbiome modifications and the association of microbiome characteristics with inflammatory parameters during the first six months of adalimumab therapy in adult patients with CD.

## 2. Materials and Methods

We performed a prospective study at the Gastroenterology Unit of “City of Health and Science of Turin”, Italy. From May 2018 to March 2019 we recruited patients: (1) affected by CD with indications to treatment with adalimumab; (2) naive to anti-TNF drugs or other biological drugs; (3) older than or equal to 18 years; (4) on a typical Mediterranean diet; (5) who agreed to sign the informed consent to participate in the study. Exclusion criteria were: (1) recent (in the last month) use of probiotic therapy; (2) recent (in the last month) use of antibiotic therapy.

### 2.1. Screening Procedures

The selected patients underwent an infectious screening (HBsAg, HBcAb, HBsAb; HCV-Ab; quantiferon-TB gold assay, chest X-ray, HIV-Ab, HPV test, VZV-Ab, EBV-Ab) before starting adalimumab therapy. Clinical history, data on physical examination, instrumental examinations, recent biochemical examinations and signed informed consent for the purpose of enrolment in the study were collected. Before starting adalimumab therapy, a faecal sample from the patients was taken, collected in the previous 24 h, in a sterile container, with the caution to reduce as much as possible contaminating contacts of the sample. The faecal samples were associated with a numerical identification code and stored frozen at −80 °C. Two faecal samples were collected from each CD patient; the first before the start of adalimumab therapy and the second after six months of treatment. The samples underwent metagenomic NGS (next generation sequencing or sequencing in parallel) sequencing with the use of the Illumina MiSeq platform (San Diego, CA, USA) following the amplification of the V3-V4 regions of the 16s-rRNA gene (ribosomal 16-S gene) using a 2 × 300 bp-end approach [9,10,11].

### 2.2. Outcomes

The primary outcome was to evaluate a possible modification of the microbiota at six months of therapy. The secondary outcomes were to evaluate: (1) the possible association of the microbiome characteristics with C-reactive protein (CRP) levels at six months of therapy; (2) the possible predictive role of the microbiome on the response to anti-TNF therapy. Response to adalimumab therapy was defined as a decrease in the Harvey-Bradshaw index (HBI) score greater than or equal to 2 (or HBI ≤ 4 at six months), in the absence of corticosteroid therapy and with adalimumab still in therapy, in agreement with the literature [12]. The study followed the principles of the Declaration of Helsinki and was approved by the local ethical committee (Comitato Etico Interaziendale A.O.U. Città della Salute e della Scienza di Torino-A.O. Ordine Mauriziano-A.S.L. Città di Torino) (approval code 0056924).

### 2.3. Statistical Analysis

Continuous variables were reported as mean ± standard error of the mean (SEM) or as median (range) depending on data distribution. The normality of the data was evaluated by D’Agostino-Pearson test. The comparison of continuous variables between independent groups was done by employing the Mann-Whitney test. The comparison of paired measurements was carried out by *t*-student test for paired measurements or by Wilcoxon test, depending on the distribution of the data. For dichotomous qualitative variables, the Chi-square test was performed. A logistic regression was performed in order to derive the odds ratio (OR), with its 95% confidence interval, as a measure of the strength of association of the two variables. A *p* value of less than 0.05 was considered significant. The statistical analysis was performed with MedCalc Statistical Software version 18.9.1 (MedCalc Software bvba, Ostend, Belgium; http://www.medcalc.org; 2018).

## 3. Results

The cohort included 20 patients. The epidemiological characteristics of the recruited patients are reported in Table 1.

Upon initiation of adalimumab therapy, 90% of patients received in combination mesalazine, 60% of patients took systemic corticosteroids and 20% took an immunosuppressant (azathioprine). Clinical, biochemical and endoscopic activity, before starting adalimumab therapy, is reported in Table 2.

### 3.1. Clinical Outcomes

**Figure 1:**
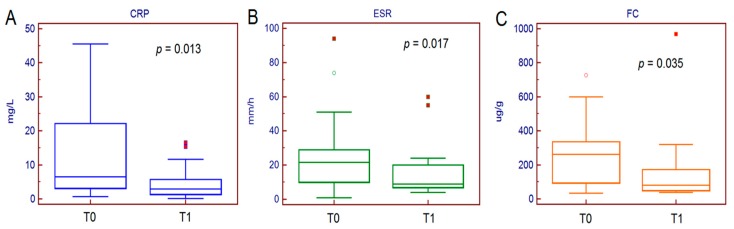
Serum and faecal inflammatory biomarkers trend after six months of adalimumab therapy.

After six months of therapy, no patient discontinued adalimumab due to adverse effects and 100% of the patients achieved clinical remission, but the success of the therapy was only achieved in 65% of patients (13 out of 20), namely the remaining seven on corticosteroid therapy. CRP decreased from a median value of 6.5 mg/L (0.7–45.5 mg/L) at T0 to a median value of 2.9 mg/L (0.1–16.5 mg/L) at T1 (*p* = 0.010). Similarly, erythrocyte sedimentation rate (ESR) decreased from the median value of 22 mm/h (1–94 mm/h) at T0 to 9 mm/h (4–60 mm/h) at T1 (*p* = 0.020). Calprotectin decreased from a median value of 262 ug/g (35–726 ug/g) at T0 to a median value of 80 ug/g (39–969 ug/g) at T1 (*p* = 0.035) (Figure 1).

### 3.2. Trend of Microbiota During Therapy

**Figure 2:**
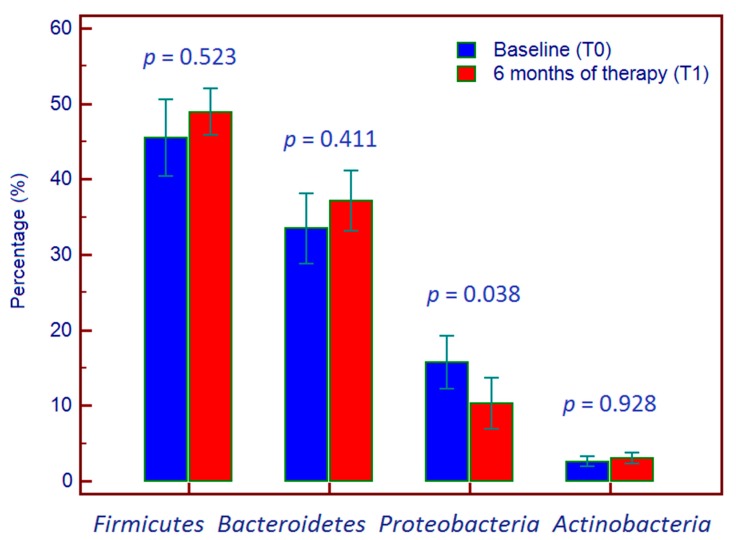
Per cent composition of phyla of bacterial microbiome at baseline and six months after starting adalimumab therapy.

Focusing on the temporal trend, regarding the phyla, *Firmicutes* rose from 45.5 ± 5.1% at T0 to 48.9 ± 3.0% at T1 (*p* = 0.523), *Bacteroidetes* from 33.5 ± 4.7% at T0 to 37.1 ± 4.0% at T1 (*p* = 0.411), *Proteobacteria* fell from 15.7% ± 3.5% at T0 to 10.3 ± 3.4% at T1 (*p* = 0.038). Finally, the *Actinobacteria* increased from 2.6% ± 0.7% at T0 to 3.0% ± 0.7% at T1 (*p* = 0.928) (Figure 2).

Regarding the bacterial families, that of *Lachnospiraceae* was the most represented both at T0 (18.2 ± 2.6%), and at T1 (23.6 ± 2.2%), without statistical difference between these two periods (*p* = 0.100). Regarding the species, *Ruminococcus gnavus* decreased from 3.3 ± 1.8% at T0 to 1.6 ± 0.3% at T1 (*p* = 0.350); *Bacteroides ovatus* rose from 2.9 ± 0.9% to 2.4 ± 0.6% (*p* = 0.540); *Faecalibacterium prausnitzii* rose from 3.7 ± 1.2% to 2.2 ± 0.8% (*p* = 0.130), *Bifidobacterium adolescentis* decreased from 1.3 ± 0.5% to 1.2 ± 0.5% (*p* = 0.260); *Escherichia coli* did not change (11.4%, *p* = 0.998).

Baseline microbiota changes in relation to success or therapeutic failure are reported in Table 3.

We also analysed the trend in composition of microbiome in relation to therapeutic success. Regarding bacterial phyla, Proteobacteria decreased in patients in whom therapeutic success was obtained, passing from a value of 15.8 ± 4.6% to 6.8 ± 3.1% (*p* = 0.049), while in non-responders, their percentage did not change (T0 = 15.6 ± 5.7% *vs.* T1 = 16.8 ± 7.6%, *p* = 0.890). The data regarding changes in composition of the microbiome in responders and in non-responders to adalimumab therapy are shown in Table 4.

In Table 5, the microbiome trend is reported according to CRP values after six months of adalimumab therapy.

With regards to the Lachnospiraceae family in patients with normalization of CRP levels after six months of adalimumab therapy, at T0 it showed a mean value of 16.6 ± 3.1% and at T1 this increased to 23.9 ± 2.6% among bacterial families (*p* = 0.049).

According to disease localization, the phylum Actinobacteria was more represented if the colon was inflamed (3.9 ± 1.0%) compared to an ileal CD (0.7 ± 0.5%); the differences among the other phyla were not statistically significant (Table S1). The changes in phyla according to disease localization were not statistically significant (Table S2).

According to disease severity, the phylum Bacteroidetes was much more represented in patients with mild or moderate endoscopic activity (41.4 ± 4.5%), compared to patients with severe endoscopic activity (15.0 ± 7.5%) (*p* = 0.006); the phylum Proteobacteria was more represented in patients with severe endoscopic activity (25.2 ± 8.6%) compared to patients with mild or moderate endoscopic activity (11.7 ± 3.0%) (*p* = 0.076) (Table S3). The changes in phyla according to endoscopic disease activity were not statistically significant (Table S4).

## 4. Discussion

It is now known that intestinal microbiota is one of the main elements capable of influencing immunity, health status, susceptibility to various diseases including chronic and autoimmune inflammatory diseases [13].

In literature, it has been reported that 25% fewer bacterial genes are detected in faecal samples of patients with IBD compared to the control groups. Furthermore, this reduction in diversity has been shown to occur early in the course of CD in a paediatric population, suggesting that dysbiosis may not only be an effect of CD, but also can contribute to the pathogenesis [14]. Further studies have shown that patients with IBD have fewer bacteria with anti-inflammatory properties and more bacteria with pro-inflammatory properties. Joossens et al. have identified stool samples containing microbiota from patients with CD with a reduced abundance of *Faecalibacterium prausnitzii*, *Bifidobacterium adolescentis* and *Dialister invisus* and a greater abundance of *Ruminococcus gnavus,* a potentially inflammatory bacterium [15]. The decrease in both biodiversity and in phyla *Bacteroidetes* and *Firmicutes* was observed in faecal and bioptic samples of patients with CD. Furthermore, many kinds of potentially protective bacteria, such as *Bacteroides*, *Eubacterium* and *Lactobacillus*, were significantly reduced in patients with active or inactive CD. *Roseburia*, a genus producing butyrate and *Phascolarctobacterium faecium* that produces propionate, have been found to be significantly reduced in patients with CD [16]. A study analysed faecal samples from a prospective cohort of patients with paediatric CD that underwent anti-TNF therapy. Regarding the dynamics of the microbiome (including viroma and micoma) with respect to therapy and diet, the dysbiosis decreased in concomitance with the reduction of the intestinal inflammation [17]. To characterise the intestinal microbiota associated with paediatric CD, Wang et al. recruited 11 children diagnosed with CD and healthy control subjects. A total of 32 samples of patients with CD were included: eight at baseline (before treatment with infliximab) and 24 at various times during therapy. Analysis of alpha diversity revealed that both wealth and diversity were lower in paediatric patients with CD before infliximab therapy compared to healthy controls. In particular, in the pre-infliximab samples a lower relative abundance of *Bacteroidetes* and a greater abundance of *Proteobacteria* were observed in patients compared to controls. After treatment, both the richness and the diversity of the intestinal microbiota improved in patients with paediatric CD. The community of bacteria in the post-infliximab samples was more similar to the control group, suggesting that the diversity between CD cases and healthy controls was reduced after treatment [18].

Changes in the composition of the faecal microbial community could therefore prove useful as biomarkers, in particular for monitoring disease activity, assessing the response to treatments [19] and as predictor of response to therapy [20,21].

In our study, we examined the relative percentage abundances of the four main bacterial phyla, namely *Firmicutes*, *Proteobacteria*, *Actinobacteria* and *Bacteroidetes*, of the family *Lachnospiraceae* and of the species *Bifidobacterium adolescentis*, *Faecalibacterium prausnitzii*, *Bacteroides Ovatus, Escherichia coli* and *Ruminococcus gnavus*. We focused on these taxa because each of them seems to have an interesting role in the pathophysiology of IBD: the *Lachnospiraceae* family (including several genera of *Clostridia* cluster XIVa, XIVb, IV and *Faecalibacterium prausnitzii*) is composed mainly of anti-inflammatory butyrogenic species and is reduced in patients with IBD, increasing proportionally to the remission of the disease [22]. *Ruminococcus gnavus* is a mucolytic bacterium found increased in IBD compared to healthy controls and is considered a possible biomarker of mucosal damage [19]. *Bifidobacteria* play a positive role in preserving intestinal barrier functions [23] and in the production of short-chain fatty acids (SCFA) [24]; of note, the analysis of the faecal microbiome of patients with IBD has shown an attenuation of *Bifidobacterium adolescentis* [25]. High antibody titres have been found targeting the antigens of *Bacteroides ovatus* [26], a bacterium that appears to be involved in the pathogenesis of IBD [27].

We assessed whether the taxa examined between the first faecal sampling and the second after six months of adalimumab therapy showed changes in terms of percentage abundance. It is interesting to note the course of the phylum *Proteobacteria* and of the family *Lachnospiraceae*. The former decreased significantly (*p* = 0.038), from 15.7 ± 3.5% to 10.3 ± 3.4%, while *Lachnospiraceae* increased from 18.2 ± 2.6% to 23.6 ± 2.2% (*p* = 0.100).

Bacterial concentrations before starting adalimumab therapy were considered in relation to achievement of therapeutic response. Although a predictive value of Firmicutes on response to therapy has been highlighted in the literature [28], and in particular of anti-inflammatory bacteria such as *Faecalibacterium prausnitzii* [20,28,29,30,31], this trend was not found in our study. Responder and non-responder patients had non-significant concentration differences of all taxa.

Then, we compared the trend of bacterial populations between T0 and T1 in those who responded versus those in whom the therapy failed. We found interesting modifications of both the phylum *Proteobacteria* and the family *Lachnospiraceae*: in those who responded to the therapy, the former decreased from T0 (15.8 ± 4.6%) to T1 (6.8 ± 3.1%) in a significant manner (*p* = 0.049). In those who did not respond to therapy, the trend was T0 = 15.6 ± 5.7%, T1 = 16.8 ± 7.6% (*p* = 0.890). With regards to the bacteria belonging to the *Lachnospiraceae* family, they increased more in responders (from 17.8 ± 3.3% to 25.4 ± 3.2%, *p* = 0.100) compared to those who did not respond (T0 = 18.8 ± 4.8%, T1 = 20.4 ± 1.8%, *p* = 0.730). With regards to the bacteria belonging to Proteobacteria phylum, *Escherichia coli* decreased from 11.4% to 4.3% (*p* = 0.078) in responders, while it remained substantially stable in those who did not respond (from 11.4% to 13.1, *p* = 0.81). Considering the trend of the intestinal microbiota during biologic therapy and the CRP values at the sixth month, there was an increase in the *Lachnospiraceae* family from T0 (16.6 ± 11%) to T1 (23.9 ± 9.6%) in patients who showed a normalization of CRP (significant: *p* = 0.049), while in those with persistent high CRP, it remained stable. The increasing trend of phylum Firmicutes and Lachnospiraceae family in patients with normalization of CRP is coherent (from 43.7–48.4% and from 16.6–23.9%, respectively): our explanation of the fact that in Lachnospiraceae family this trend is more evident is that, probably, Lachnospiraceae family, among the families belonging to phylum Firmicutes, is a species more represented in an “eubiotic” microbiota. The decrease of *Proteobacteria* and the increase of *Lachnospiraceae* is consistent with the hypothesis that adalimumab therapy, by decreasing inflammation, tends to restore the intestinal eubiosis [8,18,32].

The higher prevalence of the phylum Bacteroidetes in patients with mild or moderate endoscopic activity and the higher prevalence of the phylum Proteobacteria in patients with severe endoscopic activity confirm the potential role as protective bacteria of the former and as bacteria correlated to the inflammation of the latter.

Some limitations of our study must be discussed. The sample size of our population is not very large, although the prospective design contributes to reducing the possible biases. In all patients, diagnosis, treatment and follow-up of CD followed International Guidelines [33]. Another limit is that we focused only on some components of the human intestinal microbiota (according to literature data), even though viroma and micoma should add precious information on this topic.

## 5. Conclusions

In conclusion, in patients with CD who respond to adalimumab therapy, there is a shift of intestinal microbiome from dysbiosis closer to eubiosis. Further studies about the products of microbiota (metabolomic), and about micoma and viroma, should be performed to better understand the relationship between CD and microbiota.

## Figures and Tables

**Table 1:** Features of the study population.

General Characteristics (*n* = 20)	
Sex (M/F), *n* (%)	12/8 (60%)
Age (years), median (range)	52.5 (26–69)
Prior ileocecal resection (yes/no), *n* (%)	9/11 (45%)
Smoke (current/no), *n* (%)	4/16 (20%)
Localization (colon/ileum only), *n* (%)	12/8 (60%)
Years of illness (years), median (range)	14.5 (1–38)

Abbreviations: female (F), male (M).

**Table 2:** Activity according to Harvey-Bradshaw index (HBI) score, biochemical activity and endoscopic activity according to simple endoscopic score for Crohn’s disease (SES-CD) at baseline.

Total Patients (*n* = 20)	
Clinical activity	
Remission or mild, *n* (%)	14 (70%)
Moderate or severe, *n* (%)	6 (30%)
Biochemical activity	
CRP (mg/L), median (range)	6.5 (0.7–45.5)
ESR (mm/h), median (range) FC (µg/g), median (range)	22 (1–94) 262 (35–726)
Endoscopic activity	
Mild, *n* (%)	2 (10%)
Moderate, *n* (%)	13 (65%)
Severe, *n* (%)	5 (25%)

Abbreviations: C-reactive protein (CRP), erythrocyte sedimentation rate (ESR), faecal calprotectin (FC).

**Table 3:** Relationship between bacterial populations of phyla, family and species and therapeutic success.

	Success = Yes (%)	Success = No (%)	*p* Value
Phyla			
*Firmicutes*	45.6 ± 6.7	45.2 ± 8.4	0.960
*Bacteroidetes*	34.7± 5.3	31.1 ± 9.6	0.320
*Proteobacteria*	15.8 ± 4.6	15.6 ± 5.7	0.980
*Actinobacteria*	2.6 ± 0.8	2.6 ± 1.4	0.980
Family			
*Lachnospiraceae*	17.8 ± 3.3	18.8 ± 4.8	0.860
Species			
*Bifidobacterium adolescentis*	1.1 ± 0.5	1.6 ± 1.2	0.650
*Ruminococcus gnavus*	2.2 ± 1.0	5.5 ± 4.9	0.390
*Bacteroides ovatus*	3.9 ± 1.4	1.0 ± 0.5	0.150
*Faecalibacterium prausnitzii* *Escherichia coli*	3.6 ± 1.6 11.4 ± 4.5	3.8 ± 2.0 11.4 ± 4.9	0.940 0.998

**Table 4:** Trend in phyla, family and bacterial species according to pharmacological success or failure.

	Pharmacological Success			Pharmacological Failure		
	T0 (%)	T1 (%)	*p* Value	T0 (%)	T1 (%)	*p* Value
Phyla						
*Firmicutes*	45.6 ± 6.7	51.4 ± 3.4	0.470	45.2 ± 8.4	44.4 ± 5.8	0.900
*Bacteroidetes*	34.7 ± 5.3	38.2 ± 3.7	0.510	31.1 ± 9.6	35.2 ± 9.5	0.650
*Actinobacteria*	2.6 ± 0.8	3.2 ± 0.9	0.540	2.6 ± 1.4	2.6 ± 1.3	0.980
*Proteobacteria*	15.8 ± 4.6	6.8 ± 3.1	0.049	15.6 ± 5.7	16.8 ± 7.6	0.890
Family						
*Lachnospiraceae*	17.8 ± 3.3	25.4 ± 3.2	0.100	18.8 ± 4.8	20.4 ± 1.8	0.730
Species						
*Bifidobacterium adolescentis*	1.1 ± 0.5	1.4 ± 0.6	0.700	1.6 ± 1.2	0.7 ± 0.7	0.150
*Ruminococcus gnavus*	2.2 ± 1.0	1.8 ± 0.4	0.710	5.5 ± 4.9	1.3 ± 0.5	0.420
*Bacteroides ovatus*	3.9 ± 1.4	2.6 ± 0.6	0.240	1.0 ± 0.5	1.9 ± 1.4	0.470
*Faecalibacterium prausnitzii* *Escherichia coli*	3.6 ± 1.6 11.4 ± 4.5	2.1 ± 1.2 4.3 ± 3.1	0.080 0.078	3.8 ± 2.0 11.4 ± 4.9	2.2 ± 1.2 13.1 ± 7.7	0.540 0.812

**Table 5:** Trend of the intestinal microbiome between T0 and T1 according to C-reactive protein levels at six months.

	Normalization of CRP			Positive CRP		
	T0 (%)	T1 (%)	*p* Value	T0 (%)	T1 (%)	*p* Value
Phyla						
*Firmicutes*	43.7 ± 4.7	48.4 ± 3.2	0.290	42.2 ± 14.8	41.6 ± 8.1	0.970
*Bacteroidetes*	34.8 ± 5.9	36.9 ± 4.7	0.700	39.3 ± 10.6	48.8 ± 6.3	0.460
*Actinobacteria*	3.1 ± 1.0	3.2 ± 1.0	0.940	2.4 ± 1.4	2.4 ± 1.0	0.990
*Proteobacteria*	14.5 ± 4.4	11.4 ± 4.9	0.510	16.1 ± 7.3	2.5 ± 4.9	0.250
Family						
*Lachnospiraceae*	16.6 ± 3.1	23.9 ± 2.6	0.049	15.1 ± 1.9	16.6 ± 4.0	0.810
Species						
*Bifidobacterium adolescentis*	1.5 ± 0.7	1.0 ± 0.6	0.560	1.5 ± 0.8	0.9 ± 0.5	0.610
*Ruminococcus gnavus*	1.7 ± 0.9	1.3 ± 0.4	0.660	1.0 ± 0.5	1.9 ± 0.4	0.320
*Bacteroides ovatus*	3.2 ± 1.4	2.3 ± 0.7	0.290	1.6 ± 0.6	4.2 ± 1.9	0.260
*Faecalibacterium prausnitzii* *Escherichia coli*	3.6 ± 1.4 12.2 ± 4.3	3.0 ± 1.2 8.0 ± 4.4	0.520 0.349	6.9 ± 3.7 9.0 ± 4.0	1.0 ± 1.0 5.4 ± 4.0	0.170 0.490

## Supplementary Materials

The following are available online at https://www.mdpi.com/2077-0383/8/10/1646/s1, Table S1: Relationship between bacterial populations of phyla and disease localization, Table S2: Changes in phyla according to disease localization, Table S3: Relationship between bacterial populations of phyla and disease severity, Table S4: Changes in phyla according to disease severity.
