# Supplementary material for: Adalimumab Therapy Improves Intestinal Dysbiosis in Crohn’s Disease
Source: J Clin Med. 2019 Oct 9;8(10):1646. doi: 10.3390/jcm8101646 (PMC6832711; doi:10.3390/jcm8101646)
Supplement: Supplementary file 1 [file jcm-08-01646-s001.pdf]

Journal of
Clinical Medicine

MDPI

Table S1. Relationship between bacterial populations of phyla and disease localization.

	Colon/ileum-colon (%)	Ileum only (%)	*p* value
Phyla			
Firmicutes	50.6 ± 6.5	37.7 ± 8.0	0.224
Bacteroidetes	32.1± 6.6	35.6 ± 6.7	0.720
Actinobacteria	3.9 ± 1.0	0.7 ± 0.5	0.024
Proteobacteria	13.0 ± 5.4	19.8 ± 3.2	0.362

Table S2. Changes in phyla according to disease localization.

	Colon/ileum-colon			Ileum only		
	T0 (%)	T1 (%)	*p* value	T0 (%)	T1 (%)	*p* value
Phyla						
Firmicutes	50.6 ± 6.5	49.8 ± 3.5	0.478	37.7 ± 8.0	47.6 ± 5.8	0.184
Bacteroidetes	32.1 ± 6.6	38.4 ± 3.6	0.566	35.6 ± 6.7	35.2 ± 8.7	0.914
Actinobacteria	3.9 ± 1.0	4.1 ± 1.1	0.848	0.7 ± 0.5	1.4 ± 0.6	0.210
Proteobacteria	13.0 ± 5.4	7.4 ± 3.3	0.227	19.8 ± 3.2	14.7 ± 6.9	0.534

Table S3. Relationship between bacterial populations of phyla and disease severity.

	Mild/moderate (%)	Severe (%)	*p* value
Phyla			
Firmicutes	41.0 ± 5.4	55.9 ± 11.1	0.187
Bacteroidetes	41.4 ± 4.5	15.0 ± 7.5	0.006
Actinobacteria	2.3 ± 0.8	3.2 ± 1.6	0.571
Proteobacteria	11.7 ± 3.0	25.2 ± 8.6	0.076

Table S4. Changes in phyla according to disease severity.

	Mild/moderate			Severe		
	T0 (%)	T1 (%)	*p* value	T0 (%)	T1 (%)	*p* value
Phyla						
Firmicutes	41.0 ± 5.4	47.7 ± 3.5	0.233	55.9 ± 11.1	51.9 ± 6.9	0.772
Bacteroidetes	31.4 ± 4.5	40.2 ± 4.6	0.754	15.0 ± 7.5	30.1 ± 7.6	0.199
Actinobacteria	2.3 ± 0.8	3.1 ± 0.9	0.393	3.2 ± 1.6	2.8 ± 1.4	0.697
Proteobacteria	11.7 ± 3.0	8.4 ± 3.9	0.467	25.2 ± 8.6	14.6 ± 6.8	0.287
